# Systemic administration of urocortin after intracerebral hemorrhage reduces neurological deficits and neuroinflammation in rats

**DOI:** 10.1186/1742-2094-9-13

**Published:** 2012-01-19

**Authors:** Hock-Kean Liew, Cheng-Yoong Pang, Chih-Wei Hsu, Mei-Jen Wang, Ting-Yi Li, Hsiao-Fen Peng, Jon-Son Kuo, Jia-Yi Wang

**Affiliations:** 1 Graduate Institute of Life Sciences, National Defense Medical Center, Taipei, Taiwan; 2 Department of Medical Research, Buddhist Tzu Chi General Hospital, Hualien, Taiwan; 3 Institute of Medical Sciences, Tzu Chi University, Hualien, Taiwan; 4 Department of Emergency Medicine, Buddhist Tzu Chi General Hospital, Hualien, Taiwan; 5 School of Medicine, Tzu Chi University, Hualien, Taiwan; 6 Graduate Institute of Medical Sciences, College of Medicine, Taipei Medical University, Taipei, Taiwan

**Keywords:** anti-neuroinflammation, brain edema, intracerebral hemorrhage, urocortin

## Abstract

**Background:**

Intracerebral hemorrhage (ICH) remains a serious clinical problem lacking effective treatment. Urocortin (UCN), a novel anti-inflammatory neuropeptide, protects injured cardiomyocytes and dopaminergic neurons. Our preliminary studies indicate UCN alleviates ICH-induced brain injury when administered intracerebroventricularly (ICV). The present study examines the therapeutic effect of UCN on ICH-induced neurological deficits and neuroinflammation when administered by the more convenient intraperitoneal (i.p.) route.

**Methods:**

ICH was induced in male Sprague-Dawley rats by intrastriatal infusion of bacterial collagenase VII-S or autologous blood. UCN (2.5 or 25 μg/kg) was administered i.p. at 60 minutes post-ICH. Penetration of i.p. administered fluorescently labeled UCN into the striatum was examined by fluorescence microscopy. Neurological deficits were evaluated by modified neurological severity score (mNSS). Brain edema was assessed using the dry/wet method. Blood-brain barrier (BBB) disruption was assessed using the Evans blue assay. Hemorrhagic volume and lesion volume were assessed by Drabkin's method and morphometric assay, respectively. Pro-inflammatory cytokine (TNF-α, IL-1β, and IL-6) expression was evaluated by enzyme-linked immunosorbent assay (ELISA). Microglial activation and neuronal loss were evaluated by immunohistochemistry.

**Results:**

Administration of UCN reduced neurological deficits from 1 to 7 days post-ICH. Surprisingly, although a higher dose (25 μg/kg, i.p.) also reduced the functional deficits associated with ICH, it is significantly less effective than the lower dose (2.5 μg/kg, i.p.). Beneficial results with the low dose of UCN included a reduction in neurological deficits from 1 to 7 days post-ICH, as well as a reduction in brain edema, BBB disruption, lesion volume, microglial activation and neuronal loss 3 days post-ICH, and suppression of TNF-α, IL-1β, and IL-6 production 1, 3 and 7 days post-ICH.

**Conclusion:**

Systemic post-ICH treatment with UCN reduces striatal injury and neurological deficits, likely via suppression of microglial activation and inflammatory cytokine production. The low dose of UCN necessary and the clinically amenable peripheral route make UCN a potential candidate for development into a clinical treatment regimen.

## Background

Spontaneous intracerebral hemorrhage (ICH) accounts for approximately 15% of stroke incidents in Western populations and an even higher proportion, up to 20-30%, in Asian populations [1]. ICH is one of the most lethal and destructive types of stroke and mortality is high, at 30%-50% [2]. Despite a number of promising trials, no medical or surgical therapy has shown any benefit for ICH patients [3]. No drug increases survival in ICH patients [4]. Early surgical removal of the blood clot shows no overall benefit over more conservative therapy [5-7]. Therefore, the prognosis for ICH patients is poor.

Pathological changes in ICH can be divided into primary and secondary brain injury. Primary injury occurs rapidly as a result of physical destruction of tissues and mass expansion of the hematoma [1], and is difficult to be the therapeutic target. Secondary injury commonly occurs when the tissue reacts to blood breakdown components in the parenchyma adjacent to the hematoma, initiating a series of inflammatory responses including the activation of inflammatory cells, brain edema, blood-brain barrier (BBB) disruption and apoptosis [8]. Secondary injury often develops hours to days after the ICH insult [8], making it a practical therapeutic target. Therefore, there is still hope for using anti-inflammatory agents in ICH therapy. Urocortin (UCN) may be an ideal candidate.

UCN, a 40-amino-acid endogenous neuropeptide, belongs to the corticotrophin releasing hormone (CRH) family of peptides, which bind two G-protein coupled receptors, CRH-R1 and CRH-R2 [9,10]. These receptors are expressed in brain neurons and glial cells [11-14] in many brain regions [15], involved in the regulation of anxiety, learning and memory, body temperature, stress responses [15] and hypotension [9,16]. More importantly, UCN is considered a powerful anti-inflammatory agent by the following reports.

Intravenously administered UCN is effective in the treatment of heart ischemia/reperfusion injury [17-20]. UCN locally administered in the substantia nigra alleviates lipopolysaccharide (LPS)-induced cytotoxicity of dopaminergic neurons [21]. In our previous *in vitro *studies, we showed that UCN alleviates inflammation and neurotoxicity mediated by endotoxin-activated microglia [22,23]; while in our *in vivo *study, intracerebroventricular (ICV) treatment with UCN post-ICH reduces brain injury area, brain edema, and BBB permeability. These reductions are associated with improved neurological deficits [24]. Considering the safety and convenience of systemic administration for clinical application, we further examined the effectiveness of systemic administration of UCN in rats with experimentally induced ICH and elucidated the anti-neuroinflammatory effects of this treatment.

## Materials and methods

### Experimental design

All experimental protocols were approved by the Animal Care and Use Committee of the Tzu Chi University and National Defense Medical Center, Taiwan in accordance with guidelines set by the National Institutes of Health Guide for the Care and Use of Laboratory Animals. Animals were housed under a 12 hour light/dark cycle with free access to food and water. Utmost efforts were made to minimize the suffering and the number of animals used.

In total, 165 rats were randomly divided into the following six groups:

1. Sham + saline group (n = 17). Rats were infused with 1 μl saline into the striatum over 10 minutes, to mimic the collagenase infusion described below. At 60 minutes post-sham-ICH induction, a total of 0.2 ml of sterile saline was administered i.p. to control for UCN treatment.

2. ICH + saline group, collagenase model (n = 59). Sterile saline (0.2 ml) was administered i.p. to each animal at 60 minutes post-ICH induction by intrastriatal infusion of collagenase VII-S.

3. ICH + L-UCN group, collagenase model (n = 53). A low dose (2.5 μg/kg in 0.2 ml sterile saline, intraperitoneally) of UCN was administered to each animal at 60 minutes post-ICH.

4. ICH + H-UCN group, collagenase model (n = 24). A high dose (25 μg/kg in 0.2 ml sterile saline, i.p.) of UCN was administered to each animal at 60 minutes post-ICH.

The UCN doses were chosen according to previous studies [9,16,25-29].

5. ICH + saline group, blood infusion model (n = 6). 0.2 ml sterile saline was administered i.p. to each animal at 60 minutes post-ICH induction by intrastriatal infusion of 100 μl of autologous blood.

6. ICH + L-UCN group, blood infusion model (n = 6). A low dose (2.5 μg/kg in 0.2 ml sterile saline, i.p.) of UCN was administered to each animal at 60 minutes post-ICH

### ICH models

Male Sprague-Dawley rats (250-300 g) anesthetized with chloral hydrate (0.4 g/kg, i.p., Sigma-Aldrich, St. Louis, MO, USA). ICH was induced by stereotaxic infusion of bacterial collagenase VII-S (0.23 U in 1.0 μl sterile saline, Sigma-Aldrich) over a period of 10 minutes, or infusion of 100 μl autologous blood from the tail vein over 60 seconds, into the striatum (0.0 mm posterior, 3.0 mm right, 5.0 mm ventral to bregma at the skull surface) [30,31]. The needle was kept in place for another 10 minutes to prevent backflow. The craniotomies were sealed with bone wax. Rats were allowed to recover in separate cages at room temperature.

### Evaluation of physiological parameters

Another 22 rats were randomly assigned for evaluation of physiological parameters including mean arterial blood pressure, blood gases, body weight changes and body temperature. Under urethane (1.0 g/kg bodyweight, i.p., Sigma-Aldrich) anesthesia, a femoral artery was cannulated with a PE-50 polyethylene tube for supplementation of fluid and monitoring of arterial blood pressure and blood gas. Arterial blood pressure was recorded through an amplifier (MP35, BIOPAC system, CA, USA) and stored in a PC computer. Body temperature (rectal temperature) was automatically maintained at 37.5 ± 0.5°C by a rectal temperature sensor and a heating pad (CMA-150, Sweden). The physiological parameters were measured 10 minutes before (baseline), and 0.5, 1 and 3 hours after treatment with UCN or saline.

### Evaluation of regional Cerebral Blood Flow (rCBF)

Another 12 rats were randomly assigned for evaluation of rCBF. The rCBF was monitored with a laser probe (MNP 110XP, Oxford Optronix, UK) inserted during surgery and connected to a Laser Doppler Blood Flow Perfusion Monitor 403A (OxyFlo 2000, Oxford Optronix, UK). The probe was placed in the peri-hematomal region in the striatum (0.0 mm posterior, 5.0 mm right, 5.0 mm ventral to bregma skull surface). We collected the baseline rCBF data before and the rCBF at 0.5, 1 and 3 hours after collagenase administration. The rCBF at each time point was collected for 10 minutes and averaged. All data were normalized by the following formula: percentage change = [(*df*)/*dF*] × 100, where *df *is the mean flow after administration and *dF *is the mean flow at baseline.

### Assessment of neurological abnormalities

A total of 36 rats were used in the assessment of the neurological abnormalities by a modified Neurological Severity Score (mNSS) method [32]. The evaluation was performed by an investigator blinded to the experimental treatment scheme. The mNSS is a composite test of motor, sensory, and balance functions. The assessment was performed on day 1 before and on days 1, 3 and 7 after ICH. Neurological function was graded on a scale of 0-18 (normal score, 0; maximal deficit score, 18).

### Assessment of brain edema

Brain edema formation peaks at days 3 post-ICH [33,34]. We thus chose this time point to study brain edema, as indicated by tissue water content. A total of 35 rats were randomly used in the assessment of brain water content, using a common wet/dry method as previously described [35]. Briefly, on days 1 and 3 post-ICH, rats were anesthetized and decapitated. The brains were removed and separated into contralateral and ipsilateral hemispheres and cerebellum. The cerebellum was used as an internal control. The sample was weighed to obtain the wet weight immediately, and then dried in an oven at 100°C for 24 hours to obtain the dry weight. The water content was expressed as a percentage of the wet weight: [(wet weight)-(dry weight)] (wet weight) ^-1 ^× 100%.

### Evaluation of brain penetration of labeled UCN

Urocortin was labeled with Alexa Fluor^® ^488 dye using a Microscale Protein Labeling Kit (A30006, Invitrogen, USA), according to the manufacturer's instructions. The Alexa Fluor^® ^488 dye-labeled UCN (2.5 μg), with fluorescence excitation and emission maxima of approximately 494 and 519 nm, was administered i.p. one hour after ICH. Three hours after injection of the fluorescently labeled UCN, the rats were re-anesthetized with chloral hydrate (0.4 g/kg i.p.), and their brains were removed immediately and sectioned to 20 μm thickness with a cryostat. To confirm the entrance of urocortin peptide into the brain, 20 μm thick brain slices were labeled with anti-UCN antibody. Sections were incubated overnight at 4°C with UCN primary antibody (1:100; Catalog No. U4757, Sigma, CA, USA). The slices were then washed with PBS and incubated for 1 hour with secondary antibody (anti-rabbit-Rhodamine, Jackson ImmunoResearch, West Grove, PA, USA) at room temperature. After rinsing with PBS buffer, the slices were examined under a fluorescence microscope. After counter-staining the nuclei with DAPI, the slides were washed and mounted on cover slips with anti-fading mounting medium (VECTASHIELD^®^, CA, USA). The presence of the labeled UCN in the striatum was evaluated under a fluorescence microscope.

### Assessment of hemorrhagic volume and lesion volume

Hemoglobin content determined by spectrophotometric measurement is a good indication of the hemorrhagic volume (bleeding) on day 1 post-ICH. This volume, however, can be affected by breakdown of the hemoglobin, swelling (edema) of tissue, and production of many inflammatory mediators immediately following the bleeding. Therefore, we conducted both a spectrophotometric measurement for hemorrhagic volume and a morphometric measurement for lesion volume in the ICH + saline and ICH + L-UCN groups on days 1 and 3 post-ICH.

#### Assessment of hemorrhagic volume

Rats were randomly used in the assessment of hemorrhagic volume on day 1 (n = 6, each group) post-ICH. The accumulated hemorrhagic volume was quantified by a spectrophotometric assay as reported by Park et al., with minor modifications [36]. Briefly, both contralateral and ipsilateral hemispheres were removed after transcardial perfusion. PBS was added to the individual hemispheres to make-up a total of 3 ml volume for homogenization and centrifugation (15000 g, 30 minutes). The supernatant (40 μl) was reacted with Drabkin's reagent (160 μl, Sigma) for 15 minutes at room temperature. Optical density was measured at 540 nm with a spectrophotometer (Molecular Devices OptiMax, USA). Equivalent hemorrhage volume (μl) of the supernatant was calculated from a standard curve obtained with known amounts of blood.

#### Morphometric measurement of lesion volume

Rat brain sections were captured for morphometric measurement (image analysis) prior to being used for the cytokine ELISA assay or the dry/wet method (brain edema assay). Briefly, rat brains on day 1 (n = 6, each group) and day 3 (n = 12, each group) post-ICH were cut coronally through the needle entry plane (identifiable on the brain surface), and then serially sliced (2-mm thickness) anterior and posterior to the needle entry site. Digital photographs of the serial slices were taken and lesion volume was computed using an image analysis program (Image J, NIH). The total lesion volume (mm^3^) was computed by multiplying the blood clot area (for day 1) and lesion area (for day 3) in each section by the distance between sections [37].

### Assessment of BBB disruption with Evans blue extravasation

A total of 16 rats were randomly selected for assessment of the vascular permeability of the BBB with a modified Evans blue extravasation method [38]. Briefly, 70 hours post-ICH, rats were anesthetized with chloral hydrate (0.4 g/kg) and infused via the right femoral vein with 37°C Evans blue dye (2% in 0.9% normal saline, 4 ml/kg) over 5 minutes. Two hours later, the rats were perfused with 300 ml normal saline to wash out any remaining dye in the blood vessels and then the brains were removed and sectioned to 2 mm thickness with a rodent brain matrix. Coronal brain sections were taken starting at +2 mm and ending at -2 mm from bregma. BBB permeability was evaluated in the striatum, cortex and cerebellum. The cerebellum was used as an internal control. Each portion was weighed immediately and placed in 1 ml of 0.9% normal saline for homogenization of the sample. For protein precipitation, 1 ml of 60% trichloroacetic acid solution was added and vortexed for 2 minutes. The mixture was subsequently cooled for 30 minutes and centrifuged (1500 g at 4°C) for another 30 minutes. The absorbance of Evans blue in the supernatant was then measured with a spectrophotometer (Molecular Devices OptiMax, USA) at 610 nm. The dye concentration was expressed as μg/g of tissue weight and calculated from a standard curve obtained from known amounts of the dye.

### Assessment of cytokines

A total of 16 rats were used for the cytokine assay by ELISA. The ipsilateral striatal tissues were collected before ICH and on days 1, 3 and 7 days post-ICH. After homogenization in lysis buffer (PRO-PREP™, iNtRON Biotechnology, Korea) and centrifugation at 12,000 g for 30 minutes, the supernatants were collected and stored frozen at -80°C. During quantification, the cytokines (TNFα, IL-1β and IL-6) were normalized to 100 μg of protein in the supernatant using a commercial ELISA kit from R & D Systems (Minneapolis, MN, USA) according to the manufacturer's instructions.

### Immunohistochemistry

A total of 8 rats were used for immunohistochemistry on day 3 post-ICH. Rats were anesthetized as described above and transcardially perfused with cold 0.1 M phosphate buffer saline followed by cold 4% paraformaldehyde in 0.1 M phosphate-buffered saline. Brains were removed and immersed in 4% paraformaldehyde for 24 hours and 30% sucrose for another 24 hours. Coronal brain slices (20 μm thickness) were cut. The slices were collected at +1.0, 0.0, and -1.0 mm (center of the hemorrhagic lesion) anterior and posterior to bregma using a cryostat (Leica CM 1900). Three serial slices were taken at each plane (total 9 slices), and processed for the staining and counting of marker-specific cells [37]. Antibodies against OX-42 (1:100; Catalog No. MCA275EL, Serotec, USA) and ED-1 (1:100; Catalog No. MCA241R, Serotec, USA) were used as microglial markers, and NeuN (1:200; Catalog No. MAB377, Chemicon, USA) was used as neuron marker. Tissues sections were incubated with the primary antibodies overnight at 4°C. The bound primary antibody was visualized by incubation with an appropriate biotinylated secondary antibody followed by the Vectastain ABC reagents and color development with 3,3'-diaminobenzidine.

Negative control slices from each animal were prepared for immunohistochemical staining processed in an identical manner except the primary antibodies were omitted. OX-42^+ ^cells with dense immunoreactivity and showing a rod-like appearance were counted as activated microglia. Numbers of positive cells were counted in six squares (1 mm^2^) randomly located around the lesion in each slice. An average cell number in each plane (total 6 mm^2^) was calculated from 54 squares in 9 planes. All cell counting was done by an independent investigator.

### Statistical analysis

Data were statistically analyzed using Prism software for Student's *t*-test and are presented as mean ± standard deviation (SD). The statistical comparisons among multiple groups were made using one-way ANOVA, and multiple time points by two way ANOVA followed by Bonferroni correction. In all instances, n refers to the number of animals in a particular group. A p value of < 0.05 is considered statistically significant.

## Results

### UCN reduces neurological deficits

**Figure 1:**
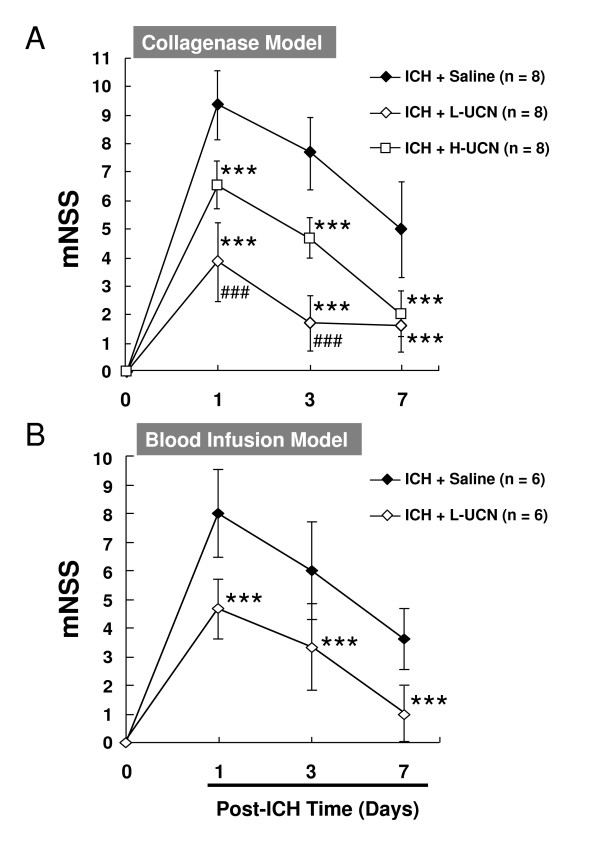
**Reduction in ICH-induced neurological deficits by UCN intraperitoneally injected post-injury**. UCN was administered i.p. at doses of 2.5 μg/kg (L) or 25 μg/kg (H) 60 minutes after ICH induced by intrastriatal injection of bacterial collagenase VII-S (A) or autologous blood infusion. (B). Modified neurological severity scores (mNSS) were examined on days 0, 1, 3, and 7 post-ICH. Values are shown as mean ± SD. ***p < 0.001 in ICH + L-UCN or ICH + H-UCN groups vs. ICH + saline group; ^###^p < 0.001 in ICH + L-UCN vs. ICH + H-UCN group. Data were analyzed as repeated measures by two-way ANOVA followed by Bonferroni correction.

L/H-UCN improved neurological deficits in a dose- and time-dependent manner in collagenase-induced ICH injury (Figure 1A). In all rats (n = 24), the mNSS was 0 before the ICH, indicating normal neurological function. In the ICH + saline (control) group (n = 8), the mNSS peaked at 9.4 ± 1.2 on day 1 and decreased to 7.7 ± 1.2 and 5.0 ± 1.7 on days 3 and 7, respectively. In the ICH + L-UCN (2.5 μg/kg) group (n = 8), the mNSS was reduced from 3.9 ± 2.6 on day 1 to 1.7 ± 2.1 and 1.6 ± 1.3 on days 3 and 7, respectively. In the ICH + H-UCN (25 μg/kg) group (n = 8), the mNSS was reduced from 6.6 ± 1.2 on day 1 to 4.7 ± 1.3 and 2.0 ± 0.8 on days 3 and 7, respectively. In addition, both L-UCN and H-UCN significantly reduced neurological deficits on days 1, 3, and 7 (p < 0.001 vs. ICH + saline group). However, the L-UCN (2.5 μg/kg) group showed a greater reduction in the neurological deficits than the H-UCN (25 μg/kg) group on days 1 and 3 (p < 0.001 vs. ICH + H-UCN group).

In the autologous blood infusion model of ICH (n = 12), we selected the dosage of UCN shown to be most effective in the collagenase model (2.5 μg/kg, i.p., n = 6) to investigate the drug's therapeutic effect (Figure 1B). The mNSS of the ICH + saline (control) group (n = 6) peaked at 8.0 ± 1.5 on day 1 and decreased to 6.5 ± 1.7 and 3.6 ± 1.1 on days 3 and 7, respectively. Similar to the findings with the collagenase model, treatment with UCN (2.5 μg/kg, n = 6) post-ICH significantly reduced the mNSS in a time-dependent manner to 4.7 ± 1.0, 3.3 ± 1.5, and 1.0 ± 1.1 on days 1, 3 and 7 (p < 0.001 vs. ICH + saline group), respectively. No animals died in any group during the experiments.

### UCN reduces brain edema

**Figure 2:**
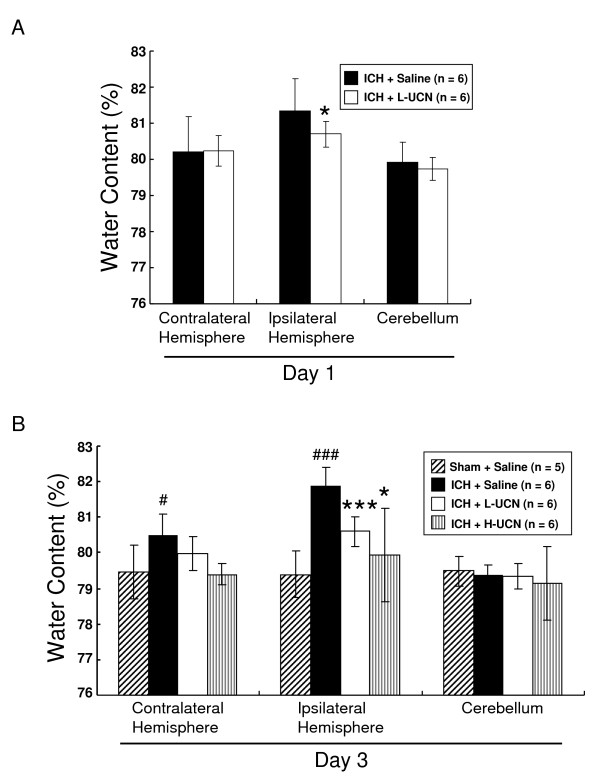
**Reduction of brain water content by post-ICH treatment with UCN**. Brain water content evaluated on day 1 (A) and 3 (B) post-ICH was expressed as percentage of the wet weight: [(wet weight)-(dry weight)] (wet weight) ^-1 ^× 100%. Values are shown as mean ± SD, calculated and analyzed by Student's t test. *p < 0.05, ***p < 0.001 vs. ICH + saline group, ^#^p < 0.05, ^### ^p < 0.001 vs. sham + saline group.

On day 1 post-ICH, UCN (ICH + L-UCN group) significantly reduced the water content of the ipsilateral hemisphere to 80.7 ± 0.3% compared to the ICH + saline group (81.4 ± 0.9%) (Figure 2A, p < 0.05). On day 3 post-ICH, water content of the contralateral hemisphere in the sham + saline group was 79.5 ± 0.8%, while that in the ICH + saline group was increased to 80.5 ± 0.6% (p < 0.05); whereas those in the ICH + L-UCN and ICH + H-UCN were insignificantly reduced to 80.0 ± 0.5% and 79.4 ± 0.3%, respectively. The water content of the ipsilateral hemisphere in the sham + saline group was 79.4 ± 0.7%, while that in the ICH + saline group was markedly increased to 81.9 ± 0.5% (p < 0.001) (Figure 2B). The water content of the ipsilateral hemispheres in both ICH + L-UCN group and ICH + H-UCN group were significantly reduced to 80.6 ± 0.4% (p < 0.001) and 79.9 ± 1.3% (p < 0.05); but the reduction in brain edema by H-UCN was not significantly different from that by L-UCN. No significant differences in water content were seen in the cerebellum between the groups (Figure 2B). These findings indicate that UCN treatment post-ICH significantly reduces the cerebral edema on day 1 and day 3.

### UCN has a hypotensive effect without changing other physiological parameters

**Figure 3:**
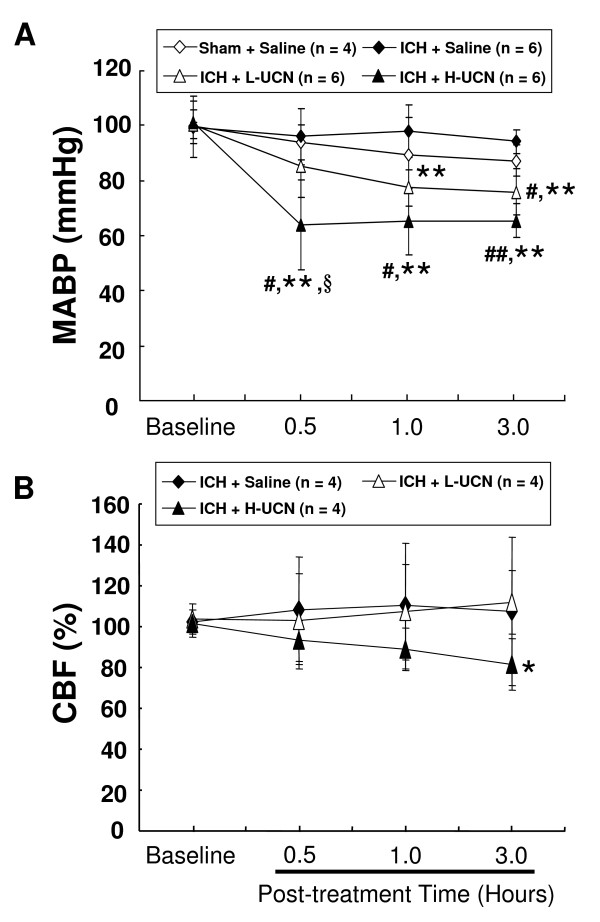
**Effects on cardiovascular function and regional cerebral blood flow induced by UCN**. (A) Mean arterial blood pressure (MABP) and (B) regional cerebral blood flow (rCBF) were measured up to 3 hours after treatment with low (L)- or high (H)-dose UCN in 4 groups of rats. Values are shown as mean ± SD. *p < 0.05, **p < 0.01 in ICH + L-UCN or ICH + H-UCN groups vs. ICH + Saline group; ^# ^p < 0.05, ^##^p < 0.001 in ICH + L-UCN or ICH + H-UCN group vs. sham + saline group; ^§^p < 0.05 in ICH + H-UCN vs. ICH + L-UCN group. Data were analyzed as repeated measures by two-way ANOVA followed by Bonferroni correction.

There were no significant differences in baseline readings of mean arterial blood pressure (MABP), heart rate (HR), rectal temperature, pO_2_, pCO_2 _and pH among the sham + saline, ICH + saline and ICH + L-UCN (2.5 μg/kg, i.p.) groups (Figure 3A & Table 1).

**Table 1:** Comparison of physiological parameters in sham + saline, ICH + saline, and ICH + L-UCN (2.5 μg/kg, i.p.) groups.

			Post-treatment Time (hours)		
			
Parameters	Grouping	Baseline	0.5	1.0	3.0
HR (beats/min)	**Sham + Saline (n = 4)**	**355.7 ± 7.0**	**378.1 ± 7.9**	**361.0 ± 29.4**	**371.3 ± 15.0**
	**ICH + Saline (n = 6)**	**349.5 ± 48.3**	**349.5 ± 41.7**	**348.8 ± 51.4**	**335.0 ± 15.9**
	**ICH + L-UCN (n = 6)**	**355.9 ± 38.3**	**356.6 ± 35.1**	376.8 ± 31.9*	389.2 ± 53.7*

RT (°C)	**Sham + Saline (n = 4)**	**37.2 ± 0.2**	**37.1 ± 0.3**	**37.1 ± 0.2**	**37.0 ± 0.2**
	**ICH + Saline (n = 6)**	**36.9 ± 0.2**	**36.9 ± 0.2**	**36.9 ± 0.1**	**35.6 ± 2.4**
	**ICH + L-UCN (n = 6)**	**36.9 ± 0.1**	**37.0 ± 0.1**	**37.0 ± 0.2**	**37.0 ± 0.1**

pO_2 _(mmHg)	**Sham + Saline (n = 4)**	**92.5 ± 2.5**	**93.6 ± 7.6**	**95.2 ± 12.6**	**102.7 ± 9.5**
	**ICH + Saline (n = 6)**	**95.7 ± 10.2**	**104.0 ± 14.5**	**104.8 ± 11.7**	**126.3 ± 29.7**
	**ICH + L-UCN (n = 6)**	**100.0 ± 8.8**	**105.2 ± 12.0**	**107.3 ± 10.7**	**107.9 ± 11.2**

pCO_2 _(mmHg)	**Sham + Saline (n = 4)**	**50.8 ± 2.9**	**50.1 ± 3.4**	**45.3 ± 1.1**	**44.4 ± 3.7**
	**ICH + Saline (n = 6)**	**49.7 ± 3.8**	**45.0 ± 3.5**	**46.2 ± 4.6**	**36.2 ± 12.6**
	**ICH + L-UCN (n = 6)**	**46.7 ± 3.6**	**45.1 ± 2.1**	**42.6 ± 4.9**	**43.3 ± 3.2**

pH	**Sham + Saline (n = 4)**	**7.39 ± 0.02**	**7.40 ± 0.03**	**7.41 ± 0.01**	**7.42 ± 0.03**
	**ICH + Saline (n = 6)**	**7.39 ± 0.03**	**7.40 ± 0.02**	**7.40 ± 0.01**	**7.39 ± 0.04**
	**ICH + L-UCN (n = 6)**	**7.37 ± 0.04**	**7.39 ± 0.02**	**7.42 ± 0.02**	**7.42 ± 0.02**

Values are shown as mean ± SD, calculated and analyzed by Student's t test. *p < 0.05 vs. ICH + saline group. ICH, intracerebral hemorrhage; HR, heart rate; L-UCN, low dose urocortin; i.p., intraperitoneally; RT, rectal temperature.

One hour after ICH, injections of L-UCN (2.5 μg/kg, i.p.) and H-UCN caused a significant decrease in MABP to a maximum of 25 mm Hg at 3.0 hours and 40 mm Hg at 0.5 hour post treatment (Figure 3A), respectively. The maximum decreases in MABP were accompanied by maximum increases in HR by 35 beats/min (L-UCN) and 45 beats/min (H-UCN) (Table 1). The rectal temperature, pO_2_, pCO_2 _and pH for the time points studied (0.5, 1, and 3 hours) were not significantly different between the groups (Table 1).

Because the low dose of UCN (2.5 μg/kg) showed better improvement in the neurological deficits in terms of reducing mNSS than the high dose (25 μg/kg), the dose of 2.5 μg/kg UCN was adopted for subsequent experiments.

### A high dose of UCN reduces the perihematomal regional cerebral blood flow (rCBF)

To clarify whether UCN affected the regional cerebral blood flow, we demonstrated that ICH alone and ICH + L-UCN did not affect perihematomal rCBF within 3 hours observation, while H-UCN significantly reduced rCBF by approximately 20% (p < 0.05, Figure 3B).

### Penetration of fluorescently labeled UCN through the barrier between blood and striatum

**Figure 4:**
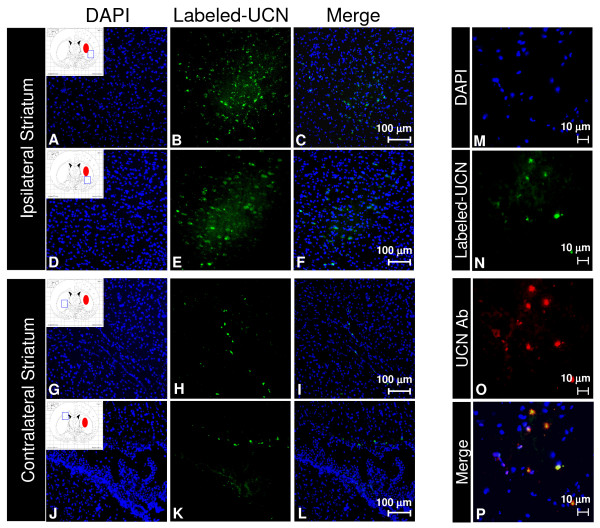
**Fluorescently labeled UCN is detected in striatal histological sections**. Alexa Fluor^® ^488-labeled-UCN was injected i.p. one hour after unilateral collagenase-ICH. The animals were sacrificed three hours after injection of the labeled-UCN. DAPI (A, D, G, J, & M blue), fluorescently labeled UCN (B, E, H, J, & M green) and total UCN (O, red) were detected by fluorescence microscopy in ipsilateral and contralateral striatum.

To examine whether UCN can be transported into the striatal parenchyma to exert its function, we administered Alexa Fluor^® ^488-labeled-UCN i.p. one hour post-ICH. The results showed that, three hours after i.p. injection of fluorescently labeled UCN (2.5 μg/kg), the labeled UCN was localized in the striatum on both ipsilateral (Figure 4A to 4F) and contralateral sides (Figure 4G to 4L). The transport of intact UCN into the brain was confirmed by dual labeling with an anti-UCN antibody, which overlaps with the Alexa Fluor^® ^488 labeled-UCN (Figure 4M to 4P). The presence of UCN appeared to be more prominent on the ipsilateral side. These results indicate that the UCN can be transported from the systemic circulation into the striatum.

### UCN reduces lesion volume but not hemorrhagic volume

**Figure 5:**
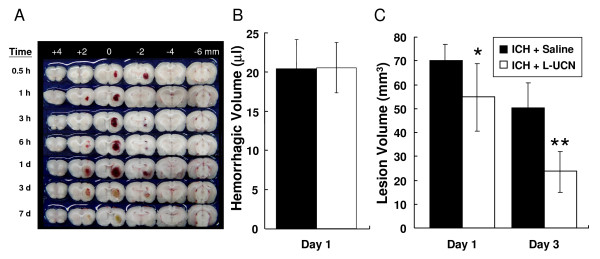
**Effects of post-ICH treatment with UCN on hemorrhagic and lesion volumes**. (A) Representative coronal sections (2 mm thickness) show brain hemorrhagic areas of seven rats killed 0.5, 1, 3, 6 hours and 1, 3, 7 days after ICH. (B) Hemorrhagic volume on day 1 (n = 6, each group) post-ICH was determined by spectrophotometric assay. (C) Lesion volume on days 1 (n = 6, each group) and 3 (n = 12, each group) post-ICH was determined by morphometric measurement. Data were analyzed as repeated measures by one-way ANOVA followed by Bonferroni correction. Values are shown as mean ± SD. *p < 0.05, **p < 0.01 vs. ICH + saline group.

The hemorrhagic area peaked on day 1 (24 hours) and declined on days 3 and 7 post-ICH (Figure 5A). The hemorrhagic volume on day 1 post-ICH for the ICH + saline and ICH + L-UCN groups were 20.4 ± 3.7 vs. 20.5 ± 3.2 μl, not significantly different between the groups (Figure 5B), indicating UCN did not affect bleeding (hemorrhagic volume). The hemorrhagic volumes for these two groups (ICH + saline vs. ICH + L-UCN group) on day 3 post-ICH were 1.3 ± 0.1 vs. 1.3 ± 0.0 μl; however, the levels were close to background, indicating complete breakdown of the hemoglobin. Therefore, lesion volume by morphometric measurement (image analysis) was used instead for days 1 and 3 post-ICH. The lesion volume was significantly reduced in ICH + L-UCN compared to the ICH + saline group on day 1 (54.7 ± 14.1 mm^3 ^vs. 70.1 ± 7.0 mm^3^, p < 0.001) and day 3 (23.7 ± 8.4 mm^3 ^vs. 50.3 ± 10.7 mm^3^, p < 0.001) (Figure 5C), suggesting that UCN can reduce lesion volume.

### UCN attenuates BBB disruption

**Figure 6:**
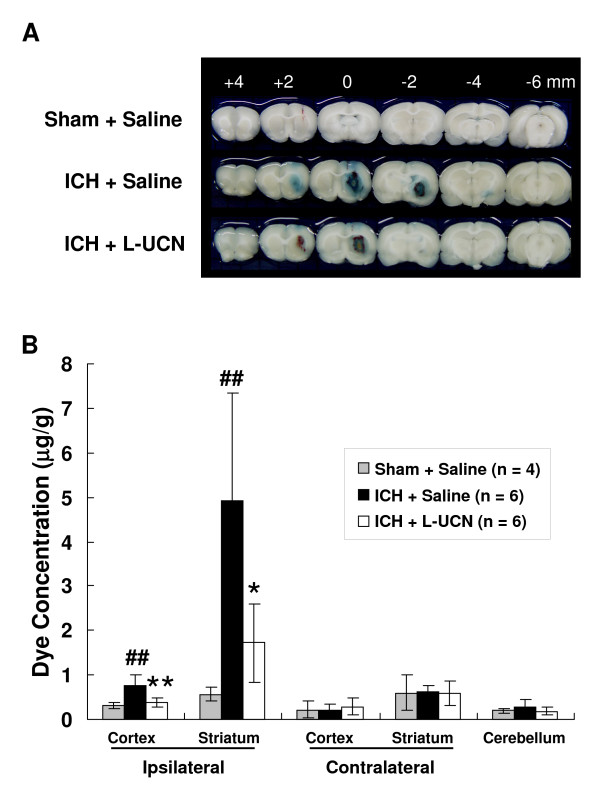
**Reduction in BBB disruption in ipsilateral cortex and striatum by post-ICH treatment with UCN**. (A) Representative brain coronal sections (2 mm thickness) show Evans blue extravasation on day 3 post-ICH. (B) Comparisons of dye concentrations in various brain tissues among sham + saline, ICH + saline, and ICH + L-UCN (2.5 μg/kg, i.p.) groups. The dye concentration is expressed as μg/g of tissue weight and calculated from a standard curve obtained from known amounts of the dye. Values are shown as mean ± SD, calculated and analyzed by Student's t test. *p < 0.05, **p < 0.01, vs. ICH + saline group, ^##^p < 0.01 vs. sham + saline group.

Since BBB disruption is very likely a contributory cause to the brain edema that peaks on day 3 post-ICH, changes in BBB disruption were determined by Evans blue dye assay on this day. Representative brain coronal sections (Figure 6A) show Evans blue extravasation on day 3 post-ICH was markedly reduced in the ICH + L-UCN group compared to the ICH + saline group. Dye concentration in the ipsilateral cortex and striatum of the ICH + saline group was significantly greater than that of the sham + saline group (0.8 ± 0.2 μg/g vs. 0.31 ± 0.1 μg/g, p < 0.01 in cortex; and 4.9 ± 2.4 μg/g vs. 0.6 ± 0.2 μg/g, p < 0.01 in striatum), indicating ICH causes BBB disruption of the ipsilateral cortex and striatum (Figure 6B). The ICH + L-UCN group exhibited a significantly lower dye concentration than the ICH + saline group in the ipsilateral cortex (0.4 ± 0.1 μg/g vs. 0.8 ± 0.2 μg/g, p < 0.01) and the striatum (1.7 ± 0.9 μg/g vs. 4.9 ± 2.4 μg/g, p < 0.05), indicating that UCN significantly reduces the ICH-induced BBB disruption. Evans blue dye concentrations in other tissues, namely cerebellum and contralateral cerebral cortex and striatum, appeared not to change in all three groups (Figure 6B).

### UCN reduces pro-inflammatory cytokine levels in striatal tissue

**Figure 7:**
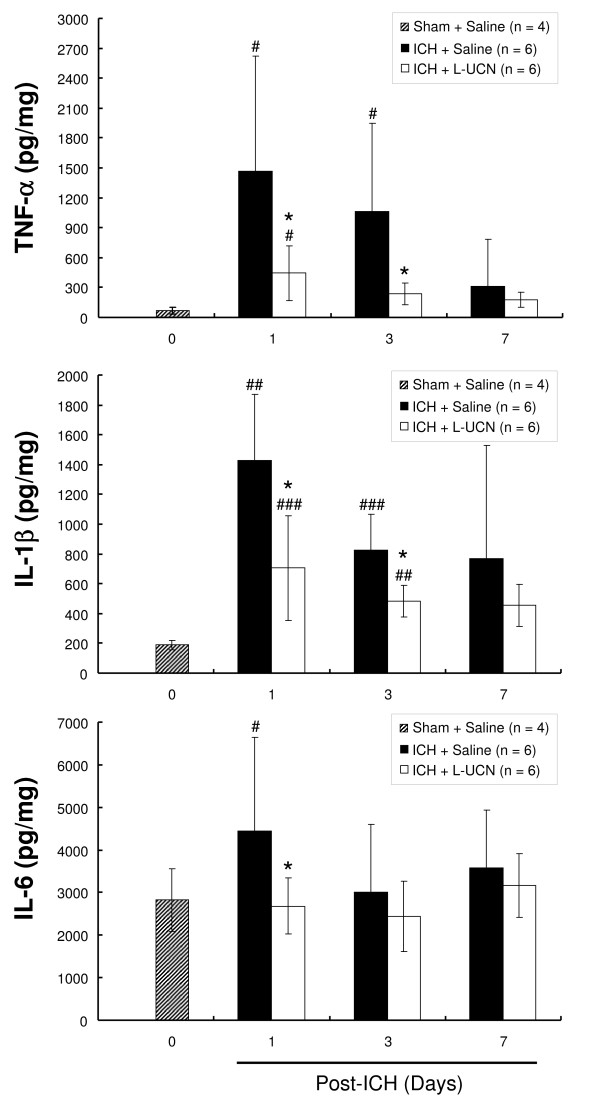
**Levels of TNF-α (A), IL-1β (B) and IL-6 (C) in striatal tissues**. After ICH, the ipsilateral striatal tissues were collected at the indicated times. The content of cytokines in the tissues was determined as described in Materials and Methods. Values are shown as mean ± SD. *p < 0.05 vs. ICH + saline group, ^#^p < 0.05, ^##^p < 0.01, ^###^p < 0.001 vs. sham + saline group. Data were analyzed as repeated measures by one way ANOVA followed by Bonferroni correction.

Pro-inflammatory cytokine (TNF-α, IL-1β, and IL-6) levels in the ipsilateral striatum in the ICH + saline group were significantly increased on day 1 post-ICH as compared with day 0 pre-ICH in the sham + saline group; levels of TNF-α (Figure 7A) and IL-1β (Figure 7B) remained high, albeit getting lower, on days 3 and 7. These high levels of cytokines were significantly reduced in the ICH + L-UCN group on days 1, 3, and 7 post-ICH, but did not return to control (normal) levels. The level of IL-6 (Figure 7C) in the ICH + saline group was also increased on day 1 post-ICH compared to the pre-ICH (day 0) level in the sham + saline group. This increase was also reduced in the ICH + L-UCN group on day 1 post-ICH. The level of IL-6, however, returned to control level without UCN treatment on days 3 and 7 post-ICH.

### UCN reduces microglial activation and neuron loss

**Figure 8:**
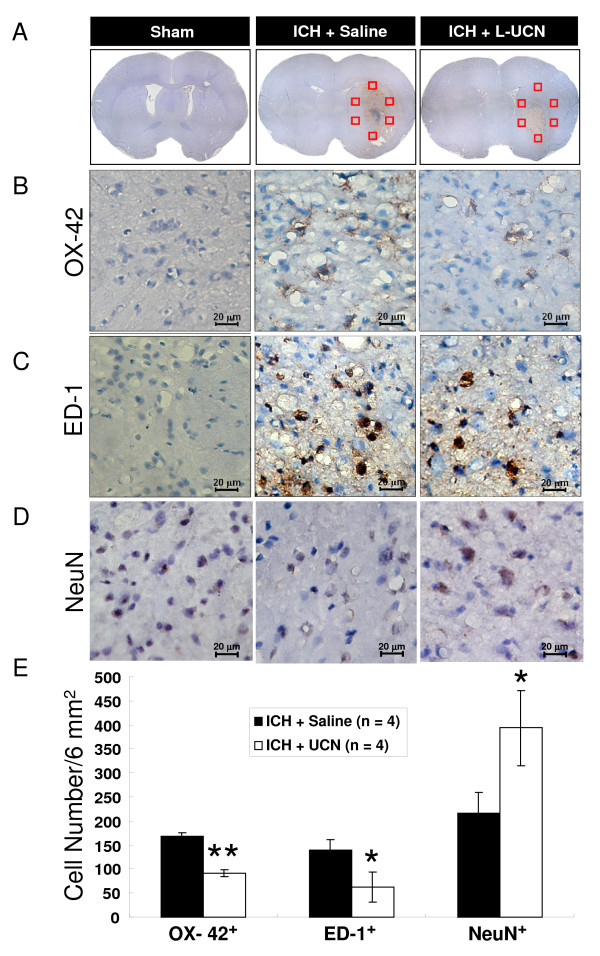
**Immunohistochemical analysis of microglial activation and neuronal loss on day 3 post-treatment with UCN**. Representative photomicrographs show: (A) low power images of peri-hematomal regions as indicated by squares; (B) high power images stained with OX-42; (C) ED-1; and (D) NeuN. (E) Average cell numbers (OX 42^+^, ED-1^+^, and NeuN^+^) in each plane (total 6 mm^2^) was calculated from 54 squares in 9 planes. Values are shown as mean ± SD, calculated and analyzed by Student's t test. *p < 0.05 vs. ICH + saline group.

When examined on day 3 post-ICH, the ICH + L-UCN group had a significantly lower number of OX-42^+ ^microglial cells (91 ± 8 cells/6 mm^2 ^vs. 168 ± 7 cells/6 mm^2^, p < 0.05, Figure 8B) and ED-1^+ ^cells (62 ± 31 cells/6 mm^2 ^vs. 140 ± 21 cells/6 mm^2^, p < 0.05, Figure 8C), as well as a significant reduction in NeuN^+ ^cell loss (393 ± 78 cells/6 mm^2 ^vs. 217 ± 42 cells/6 mm^2^, p < 0.05, Figure 8D) compared to the ICH + saline group (Figure 8E).

## Discussion

This is the first demonstration of a systemically administered very low dose of UCN offering beneficial effects in many aspects of with brain damage caused by ICH, including overall neurological function. A paradoxical dose-response was noted in which the higher dose (25 μg/kg) of UCN was less effective than the low dose (2.5 μg/kg) of UCN in attenuating neurological deficits. Brain edema leads to an increase in intracranial pressure (ICP), causing severe tissue damage. Our water content results indicate relief of edema by UCN. However, there were no significant differences between the two doses of UCN in effectively reducing brain edema, indicating the maximum effect on reduction of brain edema is reached at 2.5 μg/kg of UCN (Figure 2).

An alternative explanation of this discrepancy might be attributed to a potent hypotensive effect of UCN [9,16,25-29]. Consistent with these studies, post-treatment with high-dose UCN in our study caused a profoundly lowered MABP by 40 mmHg, which may reduce the cerebral perfusion pressure (CPP) and result in a time-dependent reduction of the striatal regional cerebral blood flow (Figure 3B) at 3 hours. Therefore, the post-treatment with high-dose UCN results in a relative hypoperfusion, which may limit the effect in reducing the brain edema and neurological deficit. Alternatively, other effects such as UCN induces the anxiogenic effect [39,40] and reduces energy expenditure [9,41] may be also factors contributing to the discrepancy of neurological improvement.

Many reports have shown that UCN is a potent appetite suppressor [9,42]. In our experiments, one bolus injection of UCN (L-UCN 2.5 μg/kg, i.p. or H-UCN 25 μg/kg, i.p) did not reduce the body weight of ICH-injured rats compared to the ICH + saline group (data not shown). The fact that a low dose is effective without causing significant untoward effects offers the advantage of a wide therapeutic safety margin, and lends support, along with the effectiveness of systemic administration, to the potential of UCN for possible clinical applications.

UCN is relatively stable in the circulation [42] and the permeability of UCN across the intact BBB is lower than other peptides and larger proteins [42]. In our study, an i.p. administration was proved to be effective. To examine whether UCN gets into the neural tissues, we injected fluorescently-labeled UCN and monitored its presence in the striatum. The results indicated appreciable uptake of intact UCN (confirmed by dual labeling with an anti-UCN antibody) in striatal tissues, most prominently in the ipsilateral hemisphere. This can be explained by the fact that several factors including leptin, glucose, insulin, and the proinflammatory cytokine and adipokine TNF-α facilitate the passage of UCN across the intact BBB [42-45]. The identification of the fluorescently labeled UCN in the injured striatum as early as 3 hours after administration may also explain the early effects of UCN in reducing the neurological deficits and inflammatory injuries (as indicated by reduced proinflammatory cytokine expression) on day 1 post-ICH.

Furthermore, we also found that intact UCN can be transported into the contralateral and ipsilateral hemispheres even in naïve brain (without sham surgery) (data not shown). The kinetics of UCN crossing the BBB into the brain were not appreciably different between the high dose and the low dose of UCN in ICH-induced injury brain. This phenomenon may be due to saturable entry of UCN through the BBB, inhibited by excess UCN [15,46]. Detailed mechanisms of UCN passage into the brain remain to be elucidated. It should however be noted that even if UCN can enter the brain, there is no proof that it functionally protects the brain at the level of the CNS. The protective effects could be mediated through peripheral effects, e.g. changes in MABP, cerebral perfusion pressure (CPP), or reduction in immune infiltration.

Glial cells (mainly astrocytes and microglia), apart from providing physical support and insulation, play important roles in maintenance and repair of neurons as well as neural transmission [47]. The cell types involved in injury and repair therefore warrant close examination. UCN was able to suppress both microglial activation and neuronal loss. These findings are compatible with UCN suppressing pro-inflammatory cytokine expression, thereby limiting inflammation and neuronal loss. Although the main role for microglial activation after brain injury is to clear cell debris and the hematoma, neuroinflammation due to over-activated microglia that release toxic factors plays a major role in further brain damage in ICH [17,18,48], especially in the early brain injury [8,48,49].

Several drugs can produce anti-oxidative effects, which protect microglia from damage, preserving their phagocytotic function for faster hematoma clearance and promotion of neuroprotection in ICH [50-52]. However, in our experiments, UCN reduced over-activated microglia that release pro-inflammatory cytokines such as TNF-α, IL-1β and IL-6, suggesting UCN has an anti-inflammatory effect. However, our results indicate no effect of UCN on hematoma clearance. This discrepancy may be due to different types of microglial activation and different types of anti-inflammatory mechanisms.

Recently, microglial activation inhibitors such as MIF (microglia/macrophage inhibitory factor, tuftsin fragment 1-3, Thr-Lys-Pro) and minocycline, have shown a promising reduction in brain edema and tissue damage, and attenuation of functional deficits in experimental ICH [49,53]. We infer that UCN is an anti-inflammatory neuropeptide that inhibits activated microglia and reduces neuronal loss *in vivo*.

Whether UCN can attenuate bleeding (hemorrhagic) volume remains a question. We found that post-ICH treatment with UCN did not affect the accumulated hemorrhagic volume at 24 hours (day 1) after UCN treatment, but significantly reduced the lesion volume on day 1 and 3 after ICH. Besides, our *in vitro *study also demonstrated that UCN (5 nM-5 μM) did not inhibit collagenase enzyme activity (data not shown); therefore, UCN may not reduce collagenase-induced bleeding. These results indicate that the neuroprotective effect of UCN may not be due to reducing bleeding volume, but most likely due to reducing brain edema and neuroinflammation.

Clinically, a stroke patient is usually admitted to the emergency clinic within 1-3 hours. The patient should be treated as early as possible, so we chose to administer UCN at one hour post-ICH. The effectiveness of the low dose of UCN is consistent with our previous *in vitro *studies, which demonstrated that femtomolar concentrations of UCN can inhibit TNF-α production in cultured microglia treated with endotoxin [22,23]. As compared with other *in vivo *anti-inflammation studies mentioned above [49,53], the effective dosage of UCN (0.5 nmol/kg, or 2.5 μg/kg, i.p.) in the present study is much more potent than MIF (≈ 1.67 μmol/kg, or 604 μg/kg) or minocycline (≈ 0.1 mmol/kg or 45 mg/kg). Therefore, UCN is a potential agent for clinically therapeutic purposes.

However, the biological activities of UCN in inflammation remain controversial. Although most studies agree that UCN is a powerful anti-inflammatory agent [22-24,54-56], some have found a pro-inflammatory effect, causing vasculitis and increased pulmonary vascular permeability [57-59]. This disparity may be due to the different subtypes of CRF receptors distributed in the CNS and periphery, and different cells and tissues, or to different effects of UCN in local and systemic administration [9,60].

UCN is a cardioprotective agent in ischemia/reperfusion-induced injuries. Activation of the PI3K/Akt and ERK 1/2 signaling pathways is part of the underlying mechanism in cardiomyocytes [17-20]. Furthermore, Abuirmeileh and colleagues infer that UCN can restore nigrostriatal functions following endotoxin-induced neuroinflammation *in vivo *[21]. We also demonstrated that the neuroprotective effect of UCN is mediated via inhibition of GSK-3β and HDAC in an *in vitro *study [22]. This current study extends the beneficial effects of UCN in the ICH rat model. However, the underlying mechanisms by which UCN reduces ICH-induced injury remain unclear and need further clarification.

## Conclusion

UCN is a potent anti-inflammatory agent that is a potential target for drug design and development for clinical use. Further investigation of UCN for clinical treatment of ICH is highly warranted.

## Competing interests

The authors declare that they have no competing interests.

## Authors' contributions

HKL performed all animal-related procedures, analyzed the data and generated the figures. CYP, MJW, JYW and CWH received funding for the project and provided input during the drafting of the manuscript. TYL assisted in evaluation of the behavioral tests and calculated cell density. HFP assessed the staining, and assisted in data collection. JSK assisted in preparation the manuscript. JYW contributed to the design and preparation of the manuscript. All authors read and approved the manuscript.
